# Relugolix in Monotherapy and Combined Therapy for the Treatment of Uterine Diseases and Its Effects on Bones: A Systematic Review

**DOI:** 10.3390/biomedicines13081851

**Published:** 2025-07-30

**Authors:** Antonio Carballo García, Ana Cristina Fernández Rísquez, Silvia Delgado García, Pablo Romero Duarte, Jesús Carlos Presa Lorite

**Affiliations:** 1 Osteoporosis Group of the Spanish Association for the Study of Menopause (AEEM), University Hospital of Jaén, 23007 Jaén, Spain; 2 Osteoporosis Group of the Spanish Association for the Study of Menopause (AEEM), Dr. Balmis University Hospital of Alicante, 03010 Alicante, Spain; 3 Osteoporosis Group of the Spanish Association for the Study of Menopause (AEEM), Poniente University Hospital of El Ejido, 04700 Almería, Spain

**Keywords:** uterine fibroids, endometriosis, menstrual bleeding, bone mineral density, relugolix, osteoporosis, gonadotropin-releasing hormone antagonists

## Abstract

**Background:** Uterine fibroids (UFs) and endometriosis are gynecological conditions that significantly increase morbidity among women of reproductive age. Relugolix, a novel gonadotropin-releasing hormone receptor antagonist, is approved in combined therapy for the management of symptoms related to these disorders. However, its potential impact on bone mineral density (BMD) and osteoporosis risk should be considered when using a gonadotropin-releasing hormone (GnRH) antagonist. This systematic review aims to evaluate the effects of daily relugolix intake in monotherapy and combination therapy on BMD, ensuring safe long-term management. **Methods:** A systematic literature review was conducted following PRISMA 2020 guidelines. Searches were performed in PubMed, Medline, and the Cochrane Library. Relevant clinical guidelines from international societies were also reviewed. Studies assessing the impact of relugolix on BMD were selected, and data on treatment efficacy, adverse effects, and bone health outcomes were synthesized. **Results:** Relugolix monotherapy has been associated with significant BMD loss due to its potent estrogen-suppressing effect. To mitigate this, combination therapy with estradiol and norethisterone acetate has been developed. Although initial monotherapy before transitioning to combination therapy results in transient BMD reduction, clinical trials have demonstrated that relugolix combination therapy maintains BMD over two years while effectively reducing endometriosis- and UF-related symptoms. **Conclusions:** Relugolix combination therapy is an effective and well-tolerated treatment for UFs and endometriosis, minimizing the risk of hypoestrogenism-related bone loss while maintaining clinical benefits. Although monotherapy may lead to transient BMD reduction, combination therapy appears to stabilize bone health.

## 1. Introduction

Uterine fibroids (UFs) are frequent benign tumors which affect the uterine muscle muscular layer, representing a significant cause of morbidity for reproductive-age women. Symptoms include heavy menstrual bleeding (HMB) and pelvic pain, among others [1]. The incidence of UFs is estimated to range between 25 and 70% [2], although the cumulative incidence in women aged 50 in the US rises to approximately 70–80% [3], thus representing the primary indication for gynecologic in-patient care among premenopausal females [1]. Despite its high prevalence and significant social and economic burden, estimated to approach billions of dollars [1,2,3,4], the etiology of this condition is not yet fully elucidated [4]. 

On the other hand, endometriosis is a gynecological disorder characterized by the presence of endometrial tissue outside the uterine cavity and commonly associated with chronic pelvic pain and infertility [5]. It affects approximately 10% of reproductive-age women and ranks as the second most common gynecological condition after UFs in the UK. This condition poses significant diagnostic and clinical challenges, with a significant number of women remaining undiagnosed for extended periods, often for years, which can lead to a decline in quality of life and disease progression [6].

Surgery has historically stood as the primary treatment for UFs, despite the high rate of re-interventions and sequelae that may occur. Indeed, it is not advisable to consider surgery as a primary treatment option for women who have not yet fulfilled their reproductive desires [2,7]. Similarly, surgical treatment of endometriosis, including laparoscopy for excision or ablation of lesions, might be effective but is often associated with recurrence rates of up to 50% within a five-year period [8]. For this reason, medical management is crucial for both conditions.

Gonadotropin-releasing hormone (GnRH) agonists, such as leuprolide acetate, have been approved for the treatment of UFs. However, these medications are associated with limitations, including the potential for rapid regrowth of fibroids following discontinuation of treatment and the occurrence of hypoestrogenic side effects, such as bone mineral density (BMD) loss. Other pharmacological strategies include aromatase inhibitors, selective progesterone receptor modulators, combined oral contraceptives, and intrauterine systems, each with varying efficacy and tolerability profiles [7]. In this context, GnRH antagonists such as elagolix, relugolix, and linzagolix have emerged as promising alternatives for the long-term, non-invasive medical management of UFs [9]. When it comes to endometriosis, medical treatment has traditionally relied on analgesics and hormonal therapies, including combined oral contraceptives and progestogens [6]. Furthermore, a comparable course has been followed in the treatment of endometriosis, for which GnRH antagonists have also recently been approved [10], thus expanding the range of therapeutic alternatives available for this indication.

GnRH antagonists such as relugolix have been shown to suppress follicle-stimulating hormone (FSH) and luteinizing hormone (LH) production in a dose-dependent manner. They also inhibit the release of ovarian steroid hormones without inducing the flare-up phenomenon [11]. The potential implications of their action for bone health are significant, since estrogen deficiency is a well-established cause of postmenopausal osteoporosis [12]. However, the incorporation of add-back therapy (E2/NETA) has been demonstrated to mitigate this symptomatology, thereby rendering this pharmaceutical group more suitable for long-term treatment applications [13].

Relugolix combination therapy (40 mg relugolix, 1mg estradiol, and 0.5 mg norethisterone acetate) was developed as a once-daily oral treatment for endometriosis-associated pain or symptomatic UFs to provide a treatment option that could be used for a longer duration. Relugolix combination therapy is also approved for management of moderate to severe pain associated with endometriosis in the US and for the symptomatic treatment of endometriosis in women with a history of previous medical or surgical treatment in the European Union and other regions. Concerning the safety profile of relugolix, no preclinical studies have been conducted with relugolix in combination with estradiol and norethisterone acetate. Nevertheless, preclinical data demonstrate that there is no particular potential hazard for humans, as evidenced by conventional studies of safety pharmacology, repeated dose toxicity, genotoxicity, and carcinogenic potential [14].

This systematic review of the literature aims to comprehensively analyze the effects of daily relugolix intake in monotherapy and in combined therapy on BMD in patients diagnosed with HMB associated with UFs and/or endometriosis. This analysis will determine the optimal approach for ensuring the safe management of the drug and evaluate the benefits of periodic densitometry controls.

## 2. Material and Methods

Studies were included if they addressed the use of relugolix for the treatment of endometriosis and/or UFs, involved female participants, included adult women of reproductive age, and were original research articles such as observational studies or clinical trials with available results.

Studies were excluded if they were duplicate records, did not involve female participants, or focused on populations not relevant to the scope of the review, such as children or individuals aged 65 years or older. Additional exclusion criteria included reviews, systematic reviews, meta-analyses, case reports, and clinical trial protocols without published results. Records that could not be retrieved or were considered off-topic after full-text assessment were also excluded from the final selection.

Following this eligibility criteria, a systematic review of the literature on the management of UFs and/or endometriosis treated with relugolix alone and in combined therapy and its effects on BMD and the risk of osteoporosis was performed in PubMed, Embase, and the Cochrane Library using the following search terms: “uterine myoma”, “uterine fibroids”, “endometriosis”, “osteoporosis”, “relugolix”, “bone mineral density”, “menopause”, “gonadotrophin-releasing hormone antagonists”. The recommendations of national and international academic societies were also considered: North American Menopause Society (NAMS), Spanish Society of Gynecology and Obstetrics (SEGO), Spanish Society for the Study of Myomas and Endometriosis (SEME), Spanish Association for the Study of Menopause (AEEM), International Menopause Society (IMS), and Canadian Menopause Society and European Menopause Society.

This systematic review has been prospectively registered in the Open Science Framework (OSF) under the following identifier: DOI 10.17605/OSF.IO/SM8JX, date: 18 July 2025.

## 3. Results

### 3.1. Workflow According to PRISMA Guidelines

**Figure 1:**
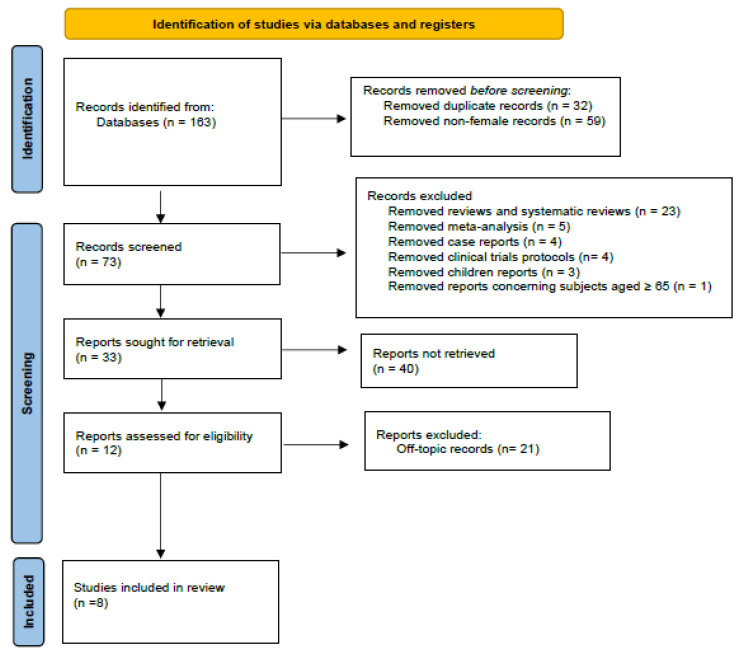
PRISMA diagram: an organized summary based on the study workflow. This figure outlines the selection procedure for the included studies.

The present study was conducted in accordance with the 2020 PRISMA guidelines [15], which supersede the 2009 statement. Studies deemed relevant to the objective were identified, selected, evaluated, and synthesized, as shown in Figure 1. A total of eight records were identified through database searches, and after the removal of duplicates and the exclusion of full-texts that did not align with the review’s criteria, the final number of records was determined.

### 3.2. Risk of Bias Assessment 

**Figure 2:**
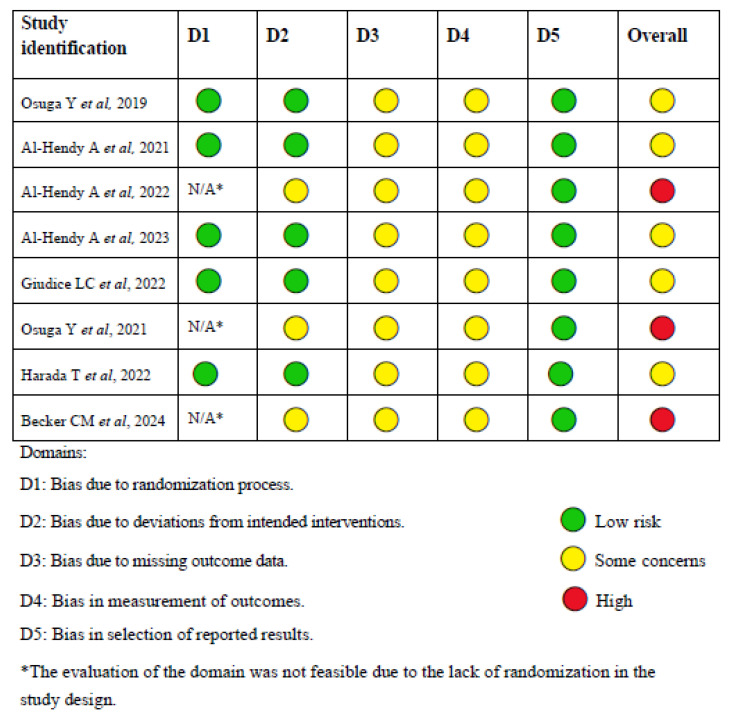
Cochrane RoB 2 tool for risk of bias [16,18,19,20,21,22,23,24].

The risk of bias was assessed using Cochrane RoB 2 tool, as shown in Figure 2. Supplementary Material Table S1 compiles a comprehensive assessment of the risk of bias across the domains, along with the underlying justifications for each domain. Additionally, it provides a global assessment of the overall risk according to the Cochrane RoB 2 criteria.

### 3.3. Overview of Research Articles

In 2019, Osuga Y et al. investigated the noninferiority of relugolix in monotherapy (40 mg, oral, once daily) compared with leuprorelin acetate (1.8 mg or 3.7 mg, monthly injection) for 6 months in reducing HMB associated with UFs in Japanese women [16]. The results demonstrated that relugolix was not only non-inferior to leuprorelin acetate (relugolix–leuprorelin difference: −0.9%; 95% CI: −10.10 to 8.35; prespecified noninferiority margin −15%; *p* = 0.001), but also induced a more rapid effect in the cessation of HMB, with adequate tolerability. The same group subsequently investigated the effect of relugolix (40 mg) over 12 weeks in reducing pain associated with UFs, finding that it resulted in an improvement in pain [17].

In the USA, Al-Handy et al. conducted a sequence of phase III trials (LIBERTY 1 and LIBERTY 2) that evaluated the efficacy and safety of relugolix combination therapy (40 mg relugolix, 1 mg E2, and 0.5 mg NETA). These trials constituted two multicenter, replicated, international, double-blind, randomized, placebo-controlled studies. The study population comprised premenopausal women between the ages of 18 and 50 years who had been diagnosed with ultrasound-confirmed fibroids and symptoms of HMB. The participants were randomly assigned to one of three groups: a 24-week placebo group; a group which received relugolix in add-back therapy for 24 weeks; or a group which received relugolix monotherapy for 12 weeks followed by add-back therapy for an additional 12 weeks [18].

The conclusion of these trials indicates that the administration of relugolix in add-back therapy once daily to women suffering from UF-related symptoms results in a significant reduction in menstrual bleeding by improving hemoglobin levels and anemia. Furthermore, the administration of relugolix in combination therapy has been demonstrated to reduce pain and discomfort related to bleeding and pelvic discomfort, preserve BMD, and minimize the occurrence of hot flashes associated with relugolix monotherapy. The most frequently observed AE was the occurrence of hot flashes. In the LIBERTY 1 study, 8% of women who received placebo, 11% of those who received relugolix in add-back therapy, and 36% of those who received relugolix in delayed add-back therapy experienced hot flashes, while in the LIBERTY 2 study, these percentages were 4% and 35%, respectively. Notably, no cases of endometrial hyperplasia or endometrial cancer were reported in the groups treated with relugolix in add-back therapy in both studies. Regarding BMD, no differences were observed between the placebo group and the group treated with relugolix add-back therapy at weeks 12 and 24. Nevertheless, in the cohort that received relugolix monotherapy for a period of 12 weeks, a progressive decrease in BMD was documented. However, subsequent to the administration of relugolix in combination with other medications for an additional 12 weeks, a stabilization of BMD was observed. The incorporation of the delayed combination therapy group in the LIBERTY trials enabled a comparative analysis of the effects of combination therapy with those of monotherapy. In both studies, it was observed that 12 weeks of treatment with monotherapy resulted in a loss of BMD and a higher incidence of vasomotor AEs compared to combination therapy. Although switching relugolix therapy to combination therapy prevented further loss of BMD, the changes in bone mass remained unaltered [18].

As reported in LIBERTY 1 and LIBERTY 2 studies [18], the overall frequency of AEs in the relugolix delayed combination therapy group was higher than in the relugolix combination therapy group and the placebo group. It is particularly noteworthy that in the relugolix delayed combination treatment group, BMD measurements in the lumbar spine and hip region demonstrated a decline following the administration of monotherapy at week 12. However, no difference between the relugolix combination treatment group and the placebo group was observed [2]. Given the similarity in BMD measurements between the relugolix combination therapy and placebo groups (at week 24, the mean percent change in bone mineral density at the lumbar spine was −0.3% with relugolix combination therapy vs. 0.0% with placebo in LIBERTY 1, and −0.1% vs. −0.2%, respectively, in LIBERTY 2; at the total hip, the changes were −0.6% vs. −0.4% in LIBERTY 1, and −0.2% vs. −0.3% in LIBERTY 2), the extension of treatment duration beyond the two-year limit is a plausible consideration. Conversely, while it is improbable that myoma size is reduced to the same extent by relugolix in combination therapy as in monotherapy, add-back therapy remains indispensable to avoid certain AEs associated with the use of the GnRH antagonist in monotherapy.

Additionally, Al-Hendy et al. [19] documented a clinical trial identified as LIBERTY 3, which encompassed 400 women suffering from UFs and who exhibited HMB. This trial constituted an extension of the LIBERTY 1 and 2 studies, using the same patient cohort. This clinical trial is characterized by its double-blind design, in which three treatment groups analogous to those employed in the LIBERTY studies were proposed. The findings from these studies indicated comparable outcomes in terms of efficacy, safety, and, specifically, BMD reduction. The conclusions drawn from these studies suggest that this treatment is effective in two primary ways: first, it can serve as a valuable alternative to surgical intervention, and second, it can optimize the patient’s symptomatology prior to surgery by reducing the size of fibroids. In addition, the studies involved the use of dual-energy X ray absorptiometry (DXA) measurements to assess the comparative safety of relugolix and add-back therapy in altering the percentage change in BMD over a 52-week period in women between the ages of 18 and 50 who were not receiving treatment. The percentage changes in BMD observed with relugolix in combination therapy through 52 weeks of treatment exhibited consistency with those reported in this cohort of age-matched women with UFs. However, BMD changes over 52 weeks showed a slight reduction in BMD in women 35 years of age or older, which were similar in relugolix-treated and naturally evolving women.

On the other hand, Al Hendy A et al. [20] conducted a study, known as LIBERTY RWS, which comprised patients who had completed the LIBERTY 3 study and opted to continue treatment for up to two years (or placebo). A total of 229 women participated in the study. These subjects were divided into two groups, with one receiving a placebo for 24 weeks followed by relugolix in combination, and the other receiving relugolix in combination for the duration of the study. The researchers measured the BMD in the total hip and femur. The study’s findings, as outlined below, are of particular relevance to the field. BMD remained stable in patients who received combination therapy for two years (mean percent change from baseline at week 104: −0.8% at lumbar spine and −0.6% at total hip). In those women who received relugolix in monotherapy for 12 weeks, BMD initially decreased, but stabilized after starting combination therapy for the remainder of the study (lumbar spine: −2.2% at week 12 and −2.3% at week 104; total hip: −1.4% at week 12 and −1.6% at week 104).

With regard to endometriosis, Giudice LC et al. [21] conducted two multicenter, randomized, double-blind, placebo-controlled, phase 3 clinical trials in 219 research centers worldwide, involving women aged 18–50 affected by this condition. The combination treatment of relugolix, administered once daily, demonstrated efficacy and reasonable tolerance in a pattern consistent with the LIBERTY studies. In regard to its effect on BMD, it was noted that BMD at the lumbar spine and total hip underwent a substantial decrease at week 12 with monotherapy using relugolix. This decline stabilized with the transition to treatment involving add-back therapy (mean percent change from baseline at lumbar spine: −2.00% at week 12 and −2.20% at week 24; at total hip: −1.30% at week 12 and −1.40% at week 24).

Osuga Y et al., which had previously assessed the effect or relugolix in UFs, found out that relugolix at doses up to 40 mg daily for 24 weeks was generally well tolerated and showed dose-dependent reductions in pelvic pain and dysmenorrhea in premenopausal Japanese women with endometriosis-associated pain. Furthermore, the 40 mg dose demonstrated comparable efficacy to leuprorelin, with a faster recovery of menstruation post-treatment and no initial hormonal flare [22]. Similar outcomes were reported by Harada et al., since once-daily 40 mg relugolix was non inferior to leuprorelin in reducing endometriosis-associated pain. Moreover, relugolix showed a faster onset of action and avoided the initial “flare-up” effect typical of GnRH agonists [23].

Finally, Becker CM et al. performed the SPIRIT open-label extension study in 2024, which showed that relugolix combined treatment over 104 weeks was well tolerated with a safety profile consistent with that observed over the first 24 weeks. It is noteworthy that after initial least squares mean BMD loss < 1% at week 24, BMD plateaued at week 36 and was sustained for the duration of 104 weeks of treatment [24].

Supplemental Material Table S2 summarizes the outcomes of the research articles included.

## 4. Discussion

The development of orally active GnRH receptor antagonists has redefined the medical management of UFs and endometriosis, which significantly impact the quality of life of reproductive-age women. They provide a prompt and reversible response with no initial hormonal flare commonly associated with GnRH agonists, representing an important advance in the long-term management of hormone-dependent gynecological conditions. Among these, elagolix was the first to demonstrate efficacy in reducing HMB in women with UFs and endometriosis. However, its high daily dose is limited to 6 months due to concerns of BMD loss. Although the lower daily dose is approved for use for up to 2 years, response rates for dysmenorrhea and non-menstrual pelvic pain were not qualitatively as high as in the group receiving the higher dose twice daily, and its effects on dyspareunia and rescue analgesic use were not statistically different from placebo [24].

As previously described, relugolix is an orally active, highly selective, non-peptidic GnRH receptor antagonist that competitively binds to pituitary GnRH receptors, thereby preventing binding to endogenous GnRH and consequently reducing the production of LH and FSH. This results in a decline in serum concentrations of E2 and P4, leading to a decrease in HMB [7,20]. However, as demonstrated in several clinical trials, relugolix monotherapy leads to significant reductions in estrogen levels, which may result in transient declines in BMD and other hypoestrogenic side effects. The addition of low-dose E2 and NETA, however, effectively mitigates these effects, stabilizing bone health during long-term treatment. In both the LIBERTY and SPIRIT trials, which evaluated women with UFs and endometriosis, respectively, combination therapy maintained BMD over 24- to 104-week treatment periods. Notably, any initial reductions observed during a monotherapy lead-in phase were stabilized upon transitioning to combination therapy [17,18,19,21,22,23].

Relugolix in combination with estradiol/norethisterone acetate (E2/NETA) add-back therapy was approved by the FDA in 2021 as the first drug administered once daily to control fibroid-related HMB in premenopausal women. The administration of add-back therapy serves to overcome the AEs resulting from low estrogen levels, thereby preventing the decrease in BMD during the 2-year treatment period [25]. Indeed, elevated E2 levels have been observed to induce epiphyseal closure by directly acting on growing cartilage cells, thereby impeding further expansion [26]. Consequently, low-dose E2 administration is recommended to stimulate bone synthesis. NETA, an oral progestin, functions by safeguarding the uterus from the adverse endometrial consequences of unopposed E2. It is employed in the treatment of menopause and endometriosis and is advisable as add-back therapy to enhance relugolix therapy [27]. Overall, the concomitant administration of relugolix 40 mg and E2/NETA (1 mg/0.5 mg) once daily was found to be generally safe and well tolerated in healthy premenopausal women [2].

In the context of endometriosis, relugolix combination therapy has demonstrated superiority over placebo and non-inferiority to injectable GnRH agonists in alleviating pain symptoms, including dysmenorrhea, non-menstrual pelvic pain, and dyspareunia. Furthermore, the combination therapy demonstrated a favorable tolerability profile, with reduced incidence of vasomotor symptoms and preservation of BMD [21,22,23].

Despite these encouraging outcomes, some limitations must be acknowledged. Data beyond two years of continuous treatment remain limited, and further investigation is warranted to confirm the long-term skeletal safety of relugolix combination therapy, particularly in women at higher baseline risk for osteoporosis. However, it is worth noting that ongoing clinical trials are addressing this evidence gap. Specifically, the phase 3B study NCT05862272 [28] is currently recruiting premenopausal women with UFs or moderate to severe endometriosis-associated pain to evaluate the long-term effects of relugolix combination therapy on BMD. In this open-label, single-arm study, participants will receive the approved daily dose of relugolix, estradiol, and NETA for up to 48 months, followed by a 12-month post-treatment follow-up period. BMD will be assessed at regular 6-month intervals using dual-energy X-ray absorptiometry (DXA). The results of this trial are expected to provide robust prospective data on the extended bone-related safety of relugolix combination therapy, contributing valuable evidence to support long-term clinical decision-making. Additionally, while the treatment has shown consistent results across diverse populations, future studies with broader demographic representation would help refine patient selection criteria and bone health monitoring protocols.

In summary, relugolix combination therapy offers an effective and well-tolerated medical treatment for hormone-dependent gynecologic conditions such as UFs and endometriosis. While monotherapy leads to transient BMD loss, combination therapy stabilizes bone health without compromising efficacy. Long-term safety data beyond two years remain limited, but current evidence supports its use as a viable alternative to surgery.

## 5. Conclusions

Relugolix combination therapy represents a promising and well-tolerated treatment option for women with endometriosis and/or UFs, effectively reducing HMB. Current evidence suggests that the addition of estradiol and norethisterone acetate to relugolix mitigates BMD loss observed with monotherapy, facilitating safer long-term management. However, transient reductions in bone density during the initial monotherapy phase highlight the need for the addition of add-back therapy. While existing clinical trials demonstrate that combination therapy effectively maintains bone health over a two-year period, additional long-term data would further strengthen our understanding of its safety profile. Continued research with extended follow-up and broader patient populations will help optimize long-term bone health monitoring strategies and support sustained therapeutic use of relugolix in combined therapy.

## Data Availability

Data are contained within the article.

## Supplementary Materials

The following supporting information can be downloaded at: https://www.mdpi.com/article/10.3390/biomedicines13081851/s1, Table S1: Risk of bias assessment; Table S2: Summary of the research articles.
