# Supplementary material for: Relugolix in Monotherapy and Combined Therapy for the Treatment of Uterine Diseases and Its Effects on Bones: A Systematic Review
Source: Biomedicines. 2025 Jul 30;13(8):1851. doi: 10.3390/biomedicines13081851 (PMC12383405; doi:10.3390/biomedicines13081851)
Supplement: Supplementary file 1 [file biomedicines-13-01851-s001.zip › biomedicines-3684194-supplementary.pdf]

Table S1. Risk of Bias Assessment.

No	Author	Domain	Risk of Bias	Justification	Overall risk
1	Osuga Y *et al*, 2019	Bias due to randomization process	Low risk	Randomized, double-blind, placebo-controlled. Block randomization used.	**Some concerns**, since the trial is judged to raise some concerns in at least one domain, but not to be at high risk of bias for any domain.
		Bias due to deviations from intended interventions	Low risk	Participants and investigators were blinded. Treatment adherence was monitored.	
		Bias due to missing outcome data	Some concerns	Not explicitly stated if missing data was handled appropriately, though all randomized patients received at least one dose.	
		Bias in measurement of outcomes	Some concerns	Self-reported NRS pain scale used; potential response bias not detailed. Analgesic use allowed.	
		Bias in selection of the reported result	Low risk	Primary and secondary outcomes were pre-specified and transparently reported.	
2	Al-Hendy A *et al*, 2021	Bias due to randomization process	Low risk	Randomized, double-blind, placebo-controlled. Stratified by menstrual blood loss volume and geographic region.	**Some concerns**, since the trial is judged to raise some concerns in at least one domain, but not to be at high risk of bias for any domain.
		Bias due to deviations from interventions	Low risk	Blinded study with identical blister packs for drug administration.	
		Bias due to missing outcome data	Some concerns	Withdrawals and dropouts reported, but missing data handling is not explicitly detailed.	
		Bias in measurement of outcomes	Some concerns	Menstrual blood loss was objectively measured, but pain scores relied on self-reports with potential subjectivity.	
		Bias in selection of reported results	Low risk	Pre-specified outcomes analyzed using a gatekeeping statistical approach to reduce reporting bias.	
3	Al-Hendy A *et al*, 2022	Bias due to randomization process	Not applicable	Open-label, single-arm extension study. No randomization involved.	**High risk**, since the trial is judged to be at high risk in at least one domain or to have some concerns for multiple domains in a way that substantially lowers confidence in the result.
		Bias due to deviations from interventions	Some concerns	Open-label study with no blinding, increasing potential for bias in subjective outcomes.	
		Bias due to missing outcome data	Some concerns	24% attrition; exclusion of patients with significant BMD loss may introduce selection bias.	
		Bias in measurement of outcomes	Some concerns	Menstrual blood loss was objectively measured, but pain and quality of life scores were self-reported, introducing subjectivity.	
		Bias in selection of reported results	Low risk	Predefined primary and secondary outcomes were reported consistently with LIBERTY 1 & 2 trials.	
4	Al-Hendy A *et al*, 2023	Bias due to randomization process	Low risk	Randomized, double-blind, placebo-controlled, stratified by baseline menstrual blood	**Some concerns,** since the trial is judged to raise some

				loss and prior relugolix exposure.	concerns in at least one domain, but not to be at high risk of bias for any domain.
		Bias due to deviations from interventions	Low risk	Blinded study with identical protocol for placebo and treatment groups.	
		Bias due to missing outcome data	Some concerns	76.7% completion rate; some discontinuations due to adverse events, handling of missing data not fully detailed.	
		Bias in measurement of outcomes	Some concerns	Menstrual blood loss measured objectively, but secondary endpoints relied on self-reports, introducing subjectivity.	
		Bias in selection of reported results	Low risk	Predefined analysis plan followed, and primary/secondary endpoints were transparently reported.	
5	Giudice LC *et al*, 2022	Bias due to randomization process	Low risk	Randomized, double-blind, placebo-controlled. Stratified by geographic region and time since endometriosis diagnosis.	**Some concerns**, since the trial is judged to raise some concerns in at least one domain, but not to be at high risk of bias for any domain.
		Bias due to deviations from interventions	Low risk	Double-blind study: identical placebo and treatment formulations ensured blinding.	
		Bias due to missing outcome data	Some concerns	15% in SPIRIT 1 and 18% in SPIRIT 2 discontinued early. Handling of missing data not fully detailed.	
		Bias in measurement of outcomes	Some concerns	Menstrual blood loss was objectively measured, but pain and quality of life scores relied on self-reports, introducing subjectivity.	
		Bias in selection of reported results	Low risk	Predefined analysis plan followed, and primary/secondary endpoints were transparently reported.	
6	Osuga Y *et al*, 2021	Bias due to randomization process	Not applicable	Open-label extension study; no randomization involved.	**High risk**, since the trial is judged to be at high risk in at least one domain or to have some concerns for multiple domains in a way that substantially lowers confidence in the result.
		Bias due to deviations from interventions	Some concerns	Open-label study; subjective outcomes like pain scores may be influenced by lack of blinding.	
		Bias due to missing outcome data	Some concerns	Patients with treatment-emergent adverse events in the preceding study were excluded; high attrition risk.	
		Bias in measurement of outcomes	Some concerns	VAS and EHP-30 were used as patient-reported outcomes without blinding.	
		Bias in selection of reported results	Low risk	Outcomes predefined and reported consistently with the original study.	
7	Harada *et al*, 2022	Bias due to randomization process	Low risk	Randomized, double-blind, double-dummy, active-controlled. Block randomization used.	**Some concerns**, since the trial is judged to raise some concerns in at least one domain, but not to be at high risk of bias for any domain
		Bias due to deviations from interventions	Low risk	Blinding of participants and investigators maintained through double-dummy design.	
		Bias due to missing outcome data	Some concerns	Discontinuations due to adverse events reported, but	

				handling of missing data not fully detailed.	
		Bias in measurement of outcomes	Some concerns	Pelvic pain assessed via self-reported VAS; analgesic use permitted.	
		Bias in selection of reported results	Low risk	Primary and secondary outcomes were pre-specified and consistently reported.	
8	Becker CM *et al*, 2024	Bias due to randomization process	Not applicable	Open-label extension of randomized trials; no randomization in this phase.	**High risk**, since the trial is judged to be at high risk in at least one domain or to have some concerns for multiple domains in a way that substantially lowers confidence in the result
		Bias due to deviations from interventions	Some concerns	Open-label design without comparator may introduce bias in patient-reported outcomes.	
		Bias due to missing outcome data	Some concerns	37,4% of patients discontinued; reasons for discontinuation varied, including lack of efficacy and adverse events.	
		Bias in measurement of outcomes	Some concerns	Pain and function assessed by self-reported NRS and EHP-30; lack of blinding could influence subjective outcomes.	
		Bias in selection of reported results	Low risk	Predefined endpoints were used and results presented in a transparent manner.	

Table S2. Summary of the research articles.

Study	Sample	Bone health outcomes	Conclusion
Oral Gonadotropin-Releasing Hormone Antagonist Relugolix Compared With Leuprorelin Injections for Uterine Leiomyomas: A Randomized Controlled Trial (Osuga Y *et al*, 2019)	Premenopausal women with UFs and HMB.	Relugolix 40 mg daily resulted in a median BMD reduction of –1.7% at week 12 and –4.4% at week 24, similar to the leuprorelin group, reflecting the hypoestrogenic effects of both treatments.	Once-daily treatment with relugolix, demonstrated noninferiority to monthly leuprorelin for improvement of HMB at 6-12 weeks of treatment, had a more rapid effect on menstrual bleeding, and was generally well tolerated.
Treatment of Uterine Fibroid Symptoms with Relugolix Combination Therapy (Al-Hendy A *et al*, 2021)	Premenopausal women experiencing HMB due to UFs.	At week 24, relugolix combination therapy maintained BMD with mean changes from baseline of –0.31% (lumbar spine), –0.53% (total hip), and –0.29% (femoral neck), while relugolix monotherapy resulted in greater BMD loss: –2.00%, –1.95%, and –1.67%, respectively.	The combination therapy of relugolix with estradiol and NETA for 24 weeks was effective in reducing HMB in women with UFs.<br>This combination maintained BMD and was generally well tolerated.
Long-term Relugolix Combination Therapy for Symptomatic Uterine Leiomyomas (Al-Hendy A *et al*, 2022)	Premenopausal women experiencing HMB associated with UFs who had completed any treatment arm in either the LIBERTY 1 or LIBERTY 2 trials.	In women receiving continuous relugolix combination therapy for 52 weeks, lumbar spine BMD was largely preserved, with mean percent changes from baseline of –0.37% at week 12, –0.23% at week 24, –0.73% at week 36, and –0.80% at week 52.	Relugolix combination therapy administered once daily was effective in sustaining improvements in HMB and anemia, as well as in reducing the symptom burden associated with UFs over a 52-week treatment period. BMD was preserved, and no new safety concerns were identified during the extended treatment duration.
LIBERTY randomized withdrawal study: relugolix combination therapy for heavy menstrual bleeding associated with uterine fibroids (Al-Hendy A *et al*, 2023)	Premenopausal women experiencing HMB associated with UFs.	Women who continued relugolix combination therapy through week 104 showed minimal changes in lumbar spine BMD, with a mean percent change of –0.80%, while those who switched to placebo after week 52 experienced a greater loss of –3.65%.	The study demonstrated that relugolix combination therapy effectively maintained reduced menstrual blood loss over a 2-year treatment period in women with UFs.
Once daily oral relugolix combination therapy versus placebo in patients with endometriosis-associated pain: two replicate phase 3, randomised, double-blind, studies (SPIRIT 1 and 2) (Giudice LC *et al,* 2022)	Women aged 18 to 50 years with surgically or visually confirmed endometriosis, experiencing moderate to severe endometriosis-associated pain.	At week 24, the least-squares mean percentage change in lumbar spine BMD was –0.70% with relugolix combination therapy versus +0.21% with placebo in SPIRIT 1, and –0.78% versus +0.02%, respectively, in SPIRIT 2; in the delayed relugolix combination group, BMD decreased by –2.0% in SPIRIT 1 and –1.9% in SPIRIT 2.	Once-daily relugolix combination therapy significantly improved endometriosis-associated pain and was well tolerated.
Two-year efficacy and safety of relugolix combination therapy in women with endometriosis-associated pain: SPIRIT open-label extension study (Becker CM *et al,* 2024)	Women with endometriosis-associated pain who completed either SPIRIT 1 or SPIRIT 2 trials.	In the 80-week open-label extension study, lumbar spine BMD remained stable, with a mean percent change from baseline of –0.39% at week 52 and –0.49% at week 104, indicating no progressive bone loss with continued relugolix combination therapy.	Long-term relugolix combination therapy maintained reductions in endometriosis-associated pain over a 2-year treatment period. BMD remained stable after a small initial decrease (<1%), and no new safety concerns were identified during the extended treatment period.
Relugolix, an oral gonadotropin-releasing hormone (GnRH) receptor antagonist, in women with endometriosis-associated pain: phase 2 safety and efficacy 24-week results (Osuga Y *et al,* 2021)	Premenopausal Japanese women with endometriosis-associated pain who completed a prior 12-week phase 2 trial.	At week 24, the mean percent change in lumbar spine BMD was –0.2% with placebo, –1.6% with relugolix 10 mg, –2.6% with 20 mg, –4.9% with 40 mg, and –4.4% with leuprorelin, showing a dose-dependent reduction in BMD with relugolix, comparable to leuprorelin at the highest dose.	Relugolix at doses up to 40 mg daily for 24 weeks was generally well tolerated and showed dose-dependent reductions in pelvic pain and dysmenorrhea. The 40 mg dose demonstrated comparable efficacy to leuprorelin, with a faster recovery of menstruation post-treatment and no initial hormonal flare. BMD loss was dose-dependent but similar to leuprorelin at the highest dose.
Relugolix, an oral gonadotropin-releasing hormone receptor antagonist, reduces endometriosis	Japanese women with moderate to severe endometriosis-	At the end of treatment, the mean percent change in lumbar spine BMD was –4.80% in the relugolix group and –4.84% in the leuprorelin group, with	Once-daily 40 mg relugolix was non inferior to leuprorelin in reducing endometriosis-associated pain. It had a faster onset of action and avoided the initial "flare-up" effect typical of GnRH agonists. The safety profiles were comparable,

associated pain compared with leuprorelin in Japanese women: a phase 3, randomized, double-blind, noninferiority study Harada T *et al,* 2022)	associated pelvic pain.	both groups showing similar reductions in bone density over 24 weeks.	although hot flushes were more common in the relugolix group.
